# Supplementary material for: The TGF-β mimic TGM4 achieves cell specificity through combinatorial surface co-receptor binding
Source: EMBO Rep. 2024 Nov 28;26(1):218–44. doi: 10.1038/s44319-024-00323-2 (PMC11723922; doi:10.1038/s44319-024-00323-2)
Supplement: Supplementary file 1 — Appendix [file 44319_2024_323_MOESM1_ESM.pdf]

The TGF-β mimic TGM4 achieves cell specificity through combinatorial surface co-receptor binding.

APPENDIX

CONTENTS

Appendix Table S1. TGM-1 and TGM-4 binding to TβRI and TβRII as assesse by SPR. Page 2

Appendix Table S2. TGM-4:CD44 binding as assessed by ITC Page 2

Appendix Table S3. Primers used for PCR amplification of truncated TGM4 proteins D1-3 and D4-5. Page 3

Appendix Table S4. List of oligonucleotides used. Page 4

Appendix Table S5: List of software used in this study. Page 5

Appendix Table S6: List of plasmids used and generated in this study Page 6

Appendix Table S1. TGM-1 and TGM-4 binding to TβRI and TβRII as assessed by SPR

Surface	Analyte (Conc.)	Fitted Parameters[1]			
		k_{on} (M^{-1} cm^{-1})	k_{off} (s^{-1})	K_D	R_{max} (RU)
TβRI	TGM1 (31.25 -250 nM)	(5.8 ± 0.5) x 10^4	(5.2 ± 0.4) x 10^{-3}	90 ± 1 nM	259 ± 2
TβRI	TGM4 (31.25 - 250 nM)	(1.9 ± 0.1) x 10^5	(9.6 ± 0.1) x 10^{-4}	5.0 ± 0.1 nM	184 ± 5
TβRII	TGM4 (12.5 – 100 μM)	(7.2 ± 0.2) x 10^3	(0.84 ± 0.03) x 10^0	116 ± 5 μM	141 ± 2
[1]Fitted parameters were derived from kinetic analysis of a single injection series					

Appendix Table S2. TGM-4:CD44 binding as assessed by ITC

Cell	TGM4-D4/5	TGM4-D4/5	TGM1-D4/5	TGM1-D4/5
Syringe	mCD44	hCD44	mCD44	hCD44
Cell Conc. (μM)	5.5	5	15	10
Syringe Conc. (μM)	60	100	100	100
Temperature (°C)	35	35	35	35
K_D (nM)	20 (7, 41)[ab]	78 (40, 136)[ab]	30 (9, 68)[ab]	122 (73, 195)[ab]
ΔH (kcal mol^{-1})	-21.7 (-23.0, -20.5)[ab]	-12.5 (-13.4, -11.6)[ab]	-19.5 (-20.7, -18.5)[ab]	-12.8 (-13.5, -12.1)[ab]
ΔG (kcal mol^{-1})	-10.9[ab]	-10.0[ab]	-10.6[ab]	-9.7[ab]
-TΔS (kcal mol^{-1})	10.8[ab]	2.5[ab]	8.9[ab]	3.0[ab]
Stoichiometry (n)	1.2[d]	2.0[d]	0.6[d]	0.9[d]

[a] Not determined due to weak signal

[b] Uncertainty reported as 68.3% confidence interval

[c] Global fit of two replicates

[d] Number of sites determined by incompetent fraction value on sedphat; set to '1' for K_D analysis

Appendix Table S3

Primers used for PCR amplification of truncated TGM4 proteins D1-3 and D4-5.

Primer Name	5' – 3' (*Asc*I)	Native nt Sequence	Nucleotide positions	Amino acid positions
coTGM4_domain1F	**gactGGCGCGCC** gcctctggctgcatg	gacagcggct gcatg	46-63	17-21 ASGCM
coTGM4_domain4F	**gactGGCGCGCC**agat gcaagcccctggaa	agatgcaagc cactggaag	787-805	263-268 RCKPLE
	**3' — 5' (*Not*I)**	**3' — 5'**	**3' — 5'**	**3' — 5'**
coTGM4_domain3R	**gactGCGGCCGC** cgggtcggggcacttccg	aggatctggg cactttcc	786-769	262-257 RKCPDP
coTGM4_domain5R	**gactGCGGCCGC** cagggcccggattcc	tagtgtgcgaa ttcc	1266 - 1252	422-418 GIRAL

For each primer, the **gact** cap is shown in lower case bold; the restriction sites (**GG^CGCGCC** for *Asc*I and **GC^GGCCGC** for *Not*I) in upper case bold, and the sequence corresponding to the domain to be amplified in plain lower case. The third column gives the native *H. polygyrus* TGM4 nucleotide sequence (Genbank Accession number MG429739) for the same segment. The right-hand columns denote the nucleotide positions in the full-length TGM4 open reading frame to which these sequences correspond, and finally the amino acid positions in the full-length TGM4 protein. Amino acid positions are given 5' to 3' for both 5'-3' and 3'-5' primers

Appendix Table S4. List of oligonucleotides used.

SL. No.	Name	Sequence (5'-3')
1.	eGFP-N1 FP	CTGCAGTCGACGGTACCGCGGCCGCATGGGATCC CCGGT
2.	eGFP-N1 RP	CTCGAGACTTGTACAGCTCGTCCATG
3.	CD72 crispr1 FP	CACCGCGCATCTAACCATCTAGGAC
4.	CD72 crispr1 RP	AAACGTCCTAGATGGTTAGATGCGC
5.	CD72 crispr2 FP	CACCGTAGATCGTTCGAAGTCATAT
6.	CD72 crispr2 RP	AAACATATGACTTCGAACGATCTAC
7.	MRC1 crispr1 FP	CACCGGTACCGGAGGGTGCAGACAA
8.	MRC1 crispr1 RP	AAACTTGTCTGCACCCTCCGGTACC
9.	MRC1 crispr2 FP	CACCGTCGGACGGATGGCTCTGGTG
10.	MRC1 crispr2 RP	AAACCACCAGAGCCATCCGTCCGAC
11.	NRP1 CRISPR 1 FP	CACCGGTTCTGTCGCTATGACCGGC
12.	NRP1 CRISPR 1 RP	AAACGCCGGTCATAGCGACAGAACC
13.	NRP1 CRISPR 2 FP	CACCGCGGACAAATCGAGTTATCAG
14.	NRP1 CRISPR 2RP	AAACCTGATAACTCGATTTGTCCGC
15.	NRP1 CRISPR 3 FP	CACCGGGAGCGCTCTACAGACCAGT
16.	NRP1 CRISPR 3 RP	AAACACTGGTCTGTAGAGCGCTCCC
17.	Itga4 CRISPR 1 FP	CACCGGGGCGAATTGGACCAAGTGA
18.	Itga4 CRISPR 1 RP	AAACTCACTTGGTCCAATTCGCCCC
19.	Itga4 CRISPR 2 FP	CACCGAGGTTGTAGGAGTGCCCGGT
20.	Itga4 CRISPR 2RP	AAACACCGGGCACTCCTACAACCTC
21.	Itga4 CRISPR 3 FP	CACCGCACGCTGTTTGGCTACTCGG
22.	Itga4 CRISPR 3 RP	AAACCCGAGTAGCCAAACAGCGTGC
23.	TGM1D123 FP AscI	GCCAGGCGCGCCGACGATTC
24.	TGM1D123 RP NotI	GACTGCGGCCGCGGGGTCTGGGCACTTG
25.	TGM1D45 FP AscI	AGCTGGCGCGCCCGGTGTAAGCCTCTGGAG
26.	TGM1D45 FP NotI	CCATGCGGCCGCCAGTGTCCTG

Appendix Table S5: List of software used in this study.

Sl. no.	Name	Source
1.	ImageStudio	LI-COR Biosciences
2.	ImageJ FIJJI	NIH
3.	Prism V 9	Graphpad
4.	DIVA V3.2	BD biosciences
5.	FlowJo V10.9	BD biosciences
6.	Scrubber	BioLogic Software
7.	Sedphat	[40,41]
8.	NITPIC	[39]
9.	GUSSI	[42]
10.	NMRPipe	[45]
11.	NMRFAM-SPARKY	[46]

Appendix Table S6: List of plasmids used and generated in this study.

Sl. n	Name	Description	Source
1.	pSpCas9(BB)-2A-GFP	CRISPR vector	PX458; Addgene
2.	pSec-Tag-2A	Modular expression vector	V90020; Thermo Scientific™
3.	pSP503	GFP expression vector	In this study
4.	pSP504	Vector for C-terminal GFP tagging	In this study
5.	pSP505	TGM1-eGFP expression vector	In this study
6.	pSP506	TGM4-eGFP expression vector	In this study
7.	pSP508	TGM1D123-eGFP expression vector	In this study
8.	pSP509	TGM1D45-eGFP expression vector	In this study
9.	pSP510	TGM4D123-eGFP expression vector	In this study
10.	pSP511	TGM4D45-eGFP expression vector	In this study
11.	Neuropilin 1 CRISPR vectors	Neuropilin 1 CRISPR vectors	In this study
12.	CD49d CRISPR vectors	CD49d CRISPR vectors	In this study
13.	CD72 CRISPR vectors	CD72 CRISPR vectors	In this study
14.	CD206 CRISPR vectors	CD206 CRISPR vectors	In this study
