# Supplementary material for: The TGF-β mimic TGM4 achieves cell specificity through combinatorial surface co-receptor binding
Source: EMBO Rep. 2024 Nov 28;26(1):218–44. doi: 10.1038/s44319-024-00323-2 (PMC11723922; doi:10.1038/s44319-024-00323-2)
Supplement: Supplementary file 3 — Expanded View Figures [file 44319_2024_323_MOESM3_ESM.pdf]

Expanded View Figures

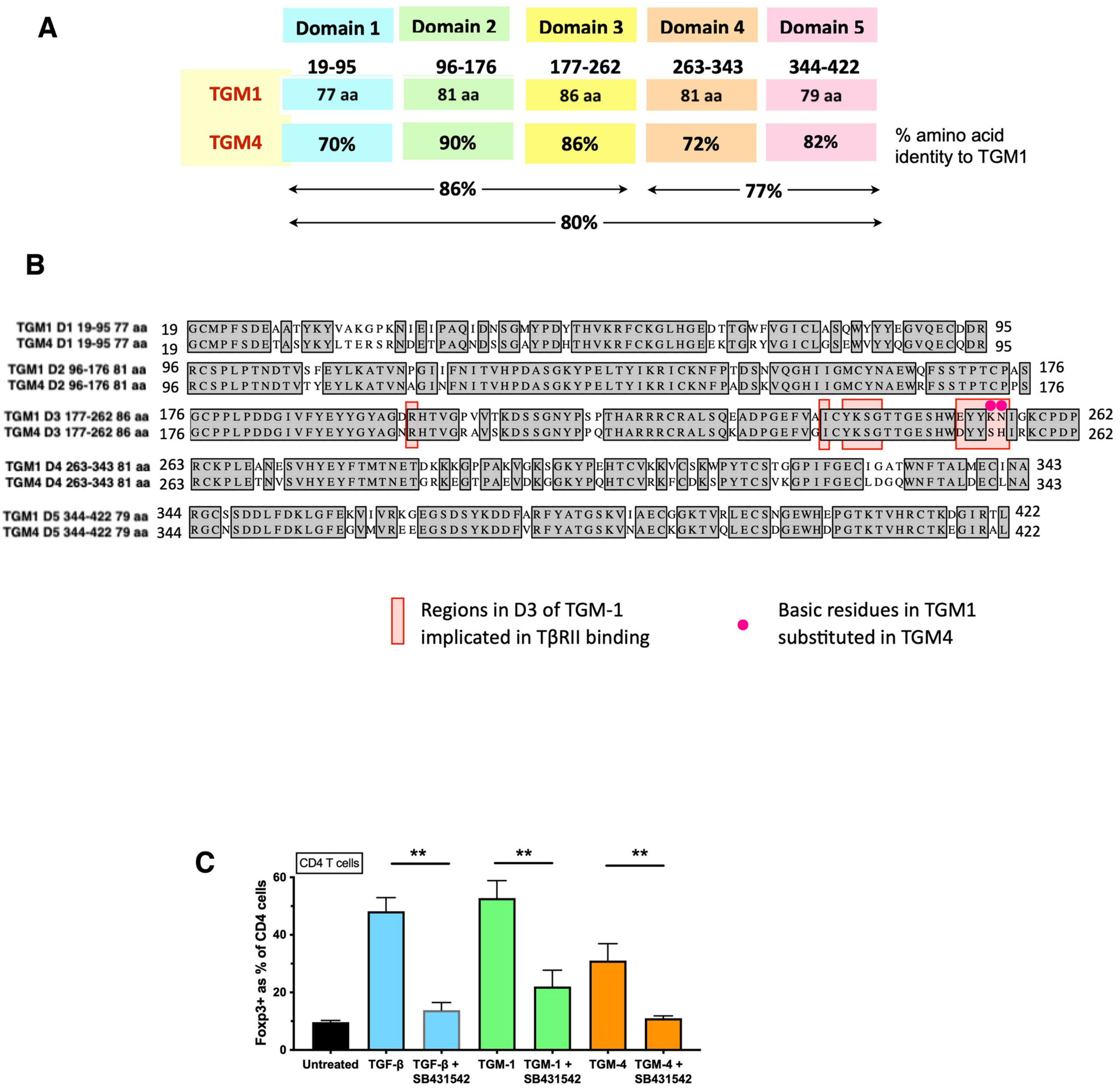

Figure EV1. Schematic structure and similarity of TGM1 and TGM4 domains.

(**A**) Schematic of domain organisation; figures denote amino acid identity for each domain to TGM1, and amino acid identity for D1-3, D4–5 and full-length TGM4. (**B**) Amino acid alignments for each domain of TGM1 and TGM4; identical domains are shaded. Red background denotes residues of TGM1 identified as contacting TβRII (Mukundan et al, 2022). (**C**) Inhibition of Foxp3 induction of TGM1 and TGM4 in the presence of SB431542, which blocks kinase activity of ALK5, receptor I for TGF-β; data represent a single experiment, $n = 3$ per group, showing mean ± SE. Data Information: Data in (**C**) analysed by unpaired t test; untreated and SB431542 treated comparisons, for TGF-β, $P = 0.0040$; for TGM1, $P = 0.0031$; for TGM4, $P = 0.0043$. $^{**}P < 0.01$.

© The Author(s)

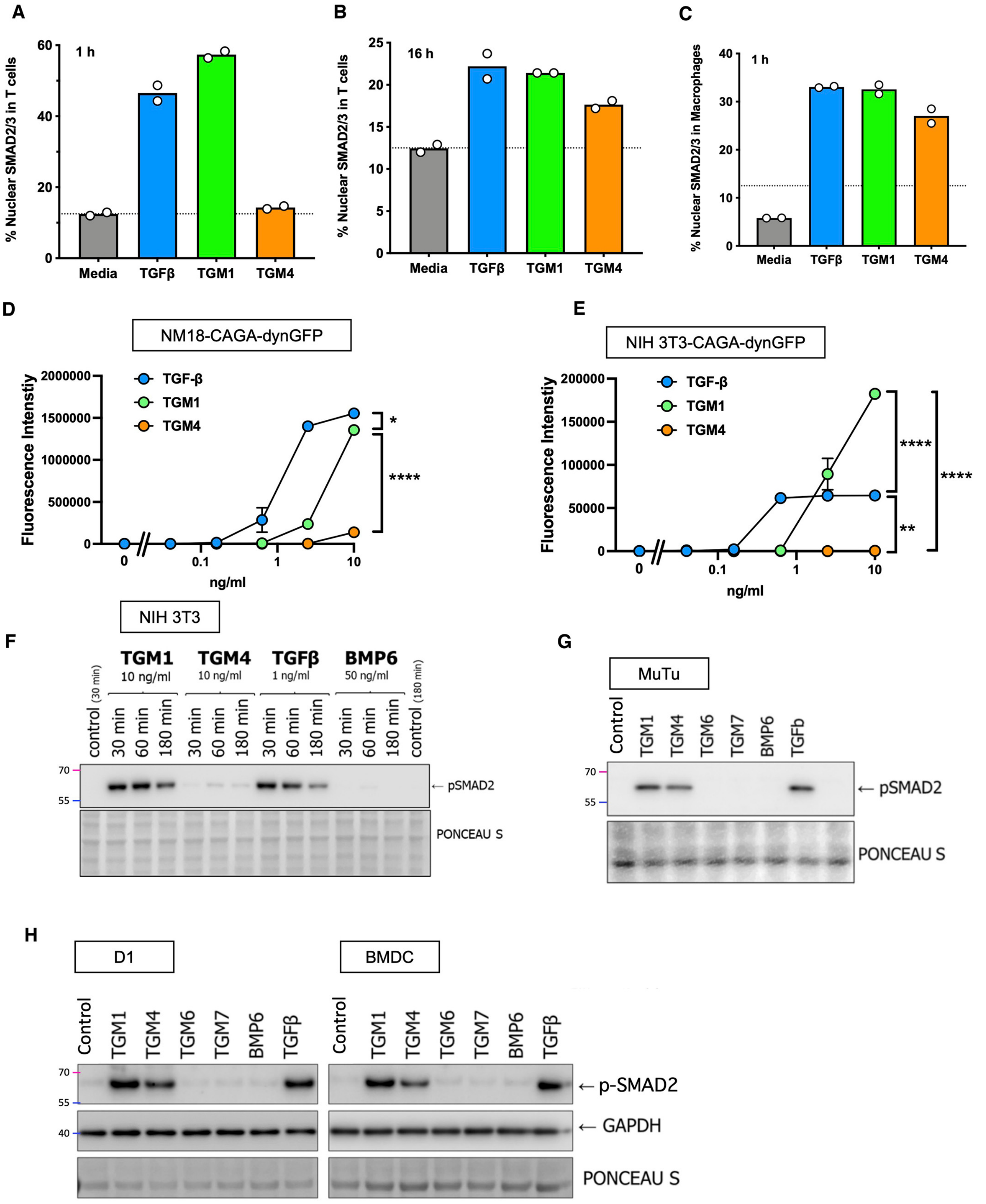
A
% Nuclear SMAD2/3 in T cells
1 h
0
20
40
60
Media
TGFβ
TGM1
TGM4
B
% Nuclear SMAD2/3 in T cells
16 h
0
5
10
15
20
25
Media
TGFβ
TGM1
TGM4
C
% Nuclear SMAD2/3 in Macrophages
1 h
0
10
20
30
40
Media
TGFβ
TGM1
TGM4
D
NM18-CAGA-dynGFP
Fluorescence Intenstiy
2000000
1500000
1000000
500000
0
TGF-β
TGM1
TGM4
*
****
0
0.1
1
10
ng/ml
E
NIH 3T3-CAGA-dynGFP
Fluorescence Intenstiy
200000
150000
100000
50000
0
TGF-β
TGM1
TGM4
****
**
****
0
0.1
1
10
ng/ml
F
NIH 3T3
TGM1 10 ng/ml
TGM4 10 ng/ml
TGFβ 1 ng/ml
BMP6 50 ng/ml
control (30 min)
30 min
60 min
180 min
control (180 min)
70
55
← pSMAD2
PONCEAU S
G
MuTu
Control
TGM1
TGM4
TGM6
TGM7
BMP6
TGFb
70
55
← pSMAD2
PONCEAU S
H
D1
BMDC
Control
TGM1
TGM4
TGM6
TGM7
BMP6
TGFβ
70
55
40
← p-SMAD2
← GAPDH
PONCEAU S

 © The Author(s)

Figure EV2. Responses of different cell types to TGM4.

(**A–C**) SMAD2/3 nuclear localisation by imaging flow cytometry in T cells at 1 h (**A**) and 16 h (**B**) post-stimulation, and in macrophages at 1 h (**C**), stimulated with TGF-β, TGM1 or TGM4, evaluated by ImageStream. Data are from one ($n = 2$) of two biological replicate experiments. (**D**, **E**) NM18 mouse mammary gland epithelial cell (**D**) and NIH 3T3 mouse embryonic fibroblast (**E**) lines transfected with the CAGA-dynGFP reporter plasmid, and stimulated with TGFβ, TGM1 or TGM4, assayed by fluorescent intensity at 24 h. Data are from one ($n = 3$) of two biological replicate experiments, presented as mean ± SE. (**F**) NIH 3T3 cells analysed for pSMAD induction by Western blot by the indicated concentrations of TGF-β, TGM1 or TGM4, for 30, 60 or 180 min. (**G**) pSMAD induction in MuTu mouse splenic dendritic cells. Cells were stimulated for one hour with 10 ng/ml of each TGM protein, 50 ng/ml BMP6 and 5 ng/ml TGF-β. (**H**) pSMAD induction in the D1 mouse dendritic cell line (left) and bone marrow-derived DCs, differentiated in vitro with GM-CSF (right). Cells were stimulated for one hour with 10 ng/ml of each TGM protein, 50 ng/ml BMP6 and 5 ng/ml TGF-β. Data Information: Data in (**D**, **E**) analysed by two-way ANOVA with Tukey's multiple comparisons test; in (**D**), at 10 ng/ml, TGF-β vs TGM1 $P = 0.0295$, and both TGF-β or TGM1 vs TGM4 $P < 0.0001$. In (**E**), TGM1 versus either TGF-β or TGM4, $P < 0.0001$; TGF-β vs TGM4, $P = 0.0053$. *$P < 0.05$, **$P < 0.01$, ****$P < 0.0001$.

© The Author(s)

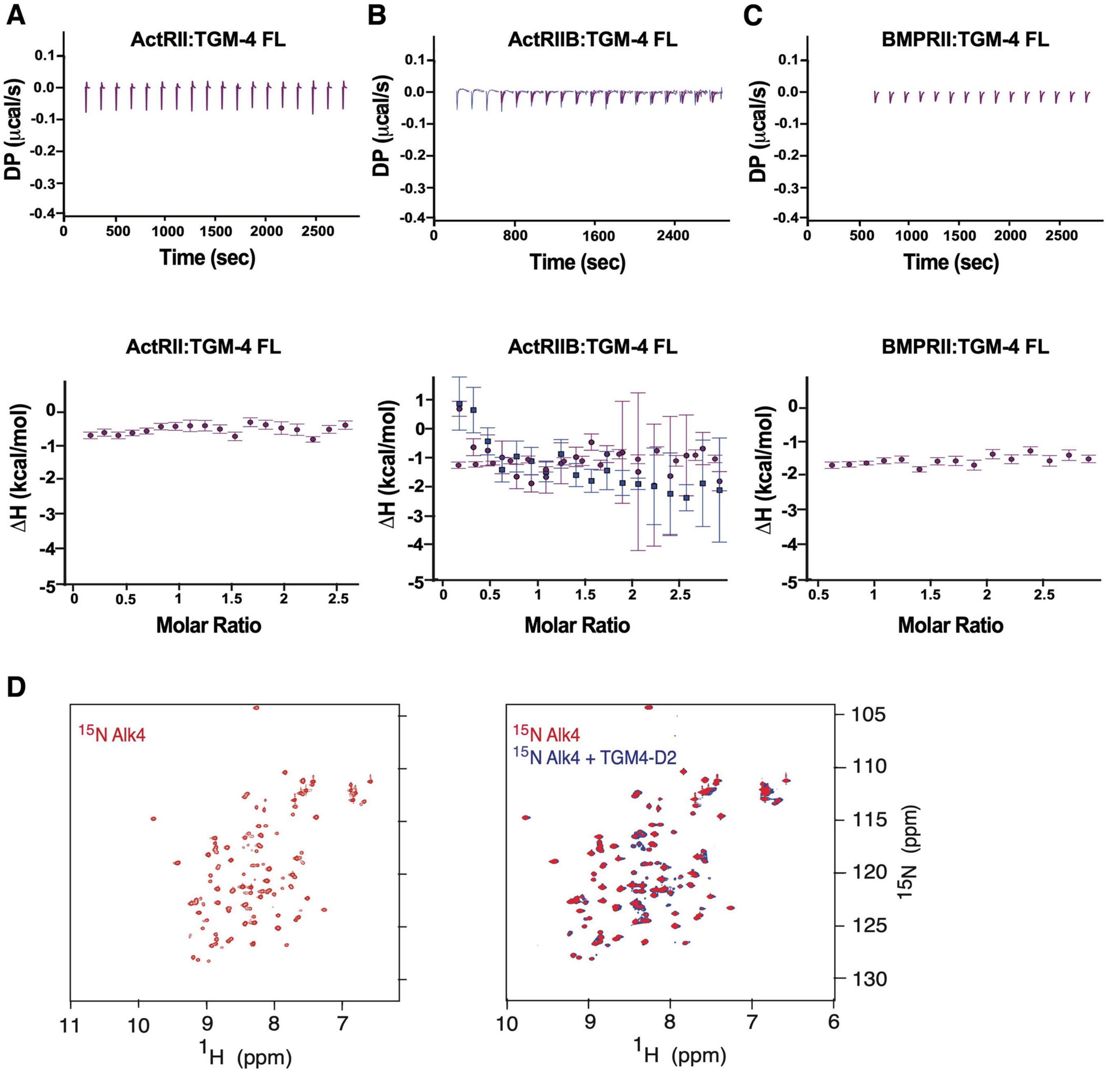

Figure EV3. Testing binding of TGM4 to other TGF-β family receptors.

(**A**) ITC analysis of interaction of ActRII with full-length (FL) TGM4; upper panel presents the raw measured heat represented as differential power (DP) for successive 2.5 μL injections of 200 μM ActRII into a cell containing 300 μL of 15 μM FL TGM4; lower panel presents the integrated heats for these data represented as the change in enthalpy (ΔH) as a function of the increasing molar ratio of ActRII to FL TGM4. One independent measurement, depicted in purple was performed. No binding isotherm could be fit. (**B**) As (**A**), for interactions of FL TGM4 with His-tagged ActRIIB; 300 μL of 10 μM FL TGM4 was titrated with successive 2.5 μL injections of 150 μM His-tagged ActRIIB). Two independent measurements, depicted in blue and purple, were performed. No binding isotherm could be fit. (**C**) As (**A**), for interactions of FL TGM4 with BMPRII; (300 μL of 10 μM FL TGM4 was titrated with successive 2.5 μL injections of 150 μM BMPRII). One independent measurement, depicted in purple was performed. No binding isotherm could be fit. (**D**) NMR analysis of TGM4 D2 interaction with ALK4 Type I receptor. ^{1}H-^{15}N spectrum of ^{15}N Alk4 alone (left, red) and overlaid onto the ^{1}H-^{15}N spectrum of ^{15}N Alk4 bound to 1.2 molar equivalents of unlabelled TGM4 D2 (right, blue).

 © The Author(s)

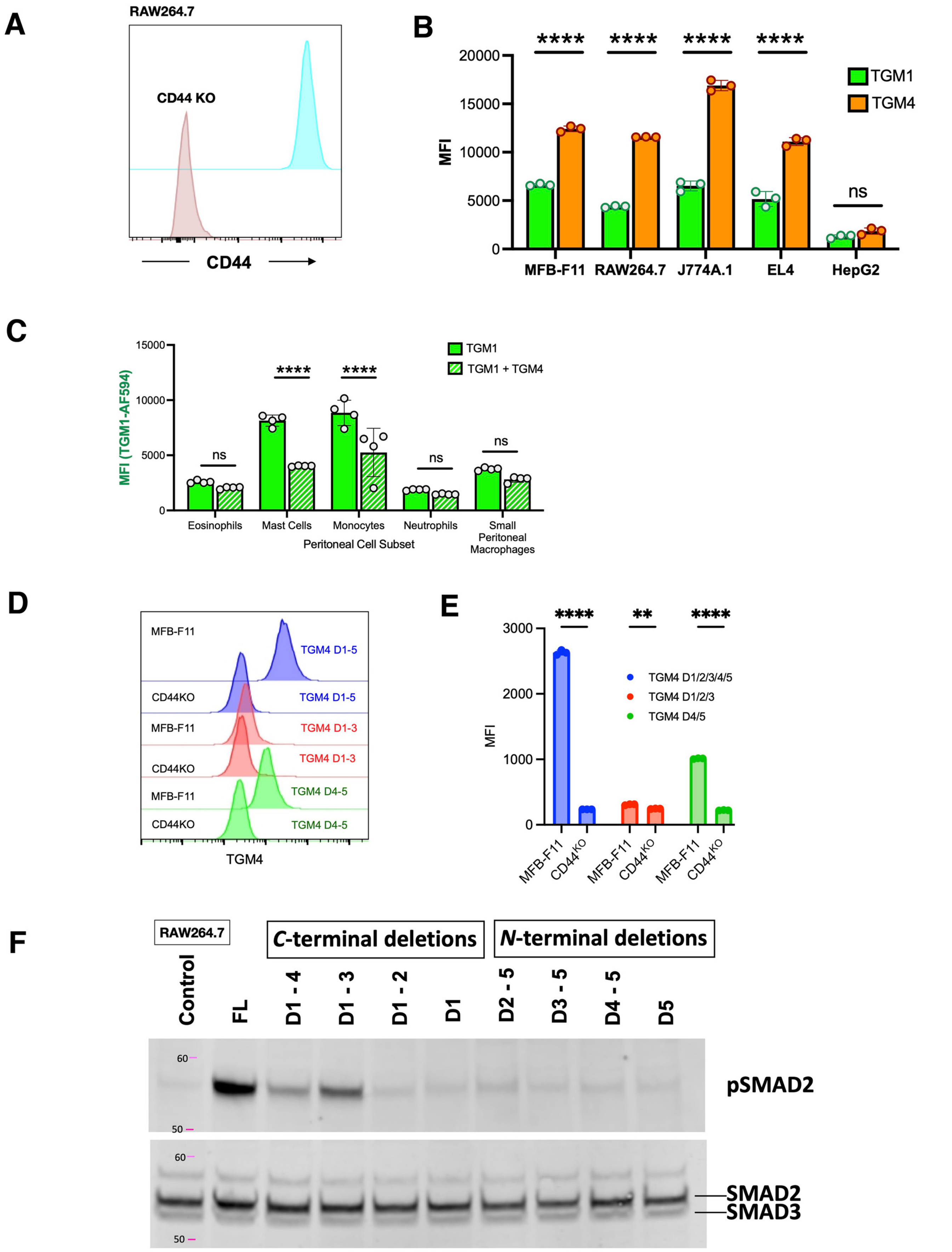
A
RAW264.7
CD44 KO
CD44
B
20000
15000
10000
5000
0
MFI
****
ns
TGM1
TGM4
MFB-F11
RAW264.7
J774A.1
EL4
HepG2
C
15000
10000
5000
0
MFI (TGM1-AF594)
****
ns
TGM1
TGM1 + TGM4
Eosinophils
Mast Cells
Monocytes
Neutrophils
Small Peritoneal Macrophages
Peritoneal Cell Subset
D
MFB-F11
CD44KO
TGM4 D1-5
TGM4 D1-3
TGM4 D4-5
TGM4
E
3000
2000
1000
0
MFI
****
**
TGM4 D1/2/3/4/5
TGM4 D1/2/3
TGM4 D4/5
MFB-F11
CD44KO
F
RAW264.7
C-terminal deletions
N-terminal deletions
Control
FL
D1 - 4
D1 - 3
D1 - 2
D1
D2 - 5
D3 - 5
D4 - 5
D5
60
50
pSMAD2
SMAD2
SMAD3

Figure EV4. Domain and co-receptor interactions of TGM4.

(**A**) Flow cytometric analysis of CD44 binding to control RAW264.7 cells (cyan) and *Cd44*-deleted RAW264.7 cells (tan). (**B**) Binding of AF594-labelled TGM1 and TGM4 constructs to the indicated cell lines, measured by Mean Fluorescence Intensity on a flow cytometer ($n = 3$). One of 2 biological replicate experiments, $n = 3$, presented as mean ± SD. (**C**) Quantification of staining of AF594-labelled TGM1 to peritoneal eosinophils (SiglecF^{+}, CD11b$^{(int)}$, Ly6G$^{(low)}$, CD117^{-}), mast cells (CD117^{+}, SSC$^{(high)}$), monocytes (CD11b^{+}, CD115^{+}, CD117^{-}, Ly6G^{-}, F4/80^{-}, SiglecF^{-}, MHC-II^{-}, CD3^{-}, CD19^{-}), neutrophils (CD11b^{+}, Ly6G^{+}, CD117^{-}) and small peritoneal macrophages (CD11b^{+}, CD117^{-}, Ly6G^{-}, F4/80$^{(low)}$, SiglecF^{-}, MHC-II$^{(high)}$, CD3^{-}, CD19^{-}), in absence (solid bars) or presence (hatched bars) of TGM4, as measured by MFI. One of 2 biological replicate experiments, $n = 4$, presented as mean ± SD. (**D**, **E**) Flow cytometric analysis of TGM4 binding to MFB-F11 wild-type and CD44-deficient cells, probed with full-length TGM4 D1-5, and truncated constructs D1-3 and D4–5. Example histograms (**D**) and results from 3 biological replicate experiments (**E**) are shown. Data shown ($n = 3$), presented as mean ± SD. (**F**) pSMAD induction in RAW264.7 cells by truncated 10 ng/mLTGM4 constructs, with C-terminal deletions and N-terminal deletions, assessed by western blotting. FL Full-length. Data Information: Data in (**B**, **C**, **E**), analysed by 2-way ANOVA; in (**B**), each comparison between TGM1 and TGM4, $P < 0.0001$; in (**C**), comparison between TGM1 and TGM4 for mast cells and macrophages; in (**E**), comparisons between wild-type and CD44 KO MFB-F11 fibroblasts with TGM4 D1-5 and TGM4 D4–5, both $P < 0.0001$; comparison with TGM4 D1-3, $P = 0.0084$.

 © The Author(s)

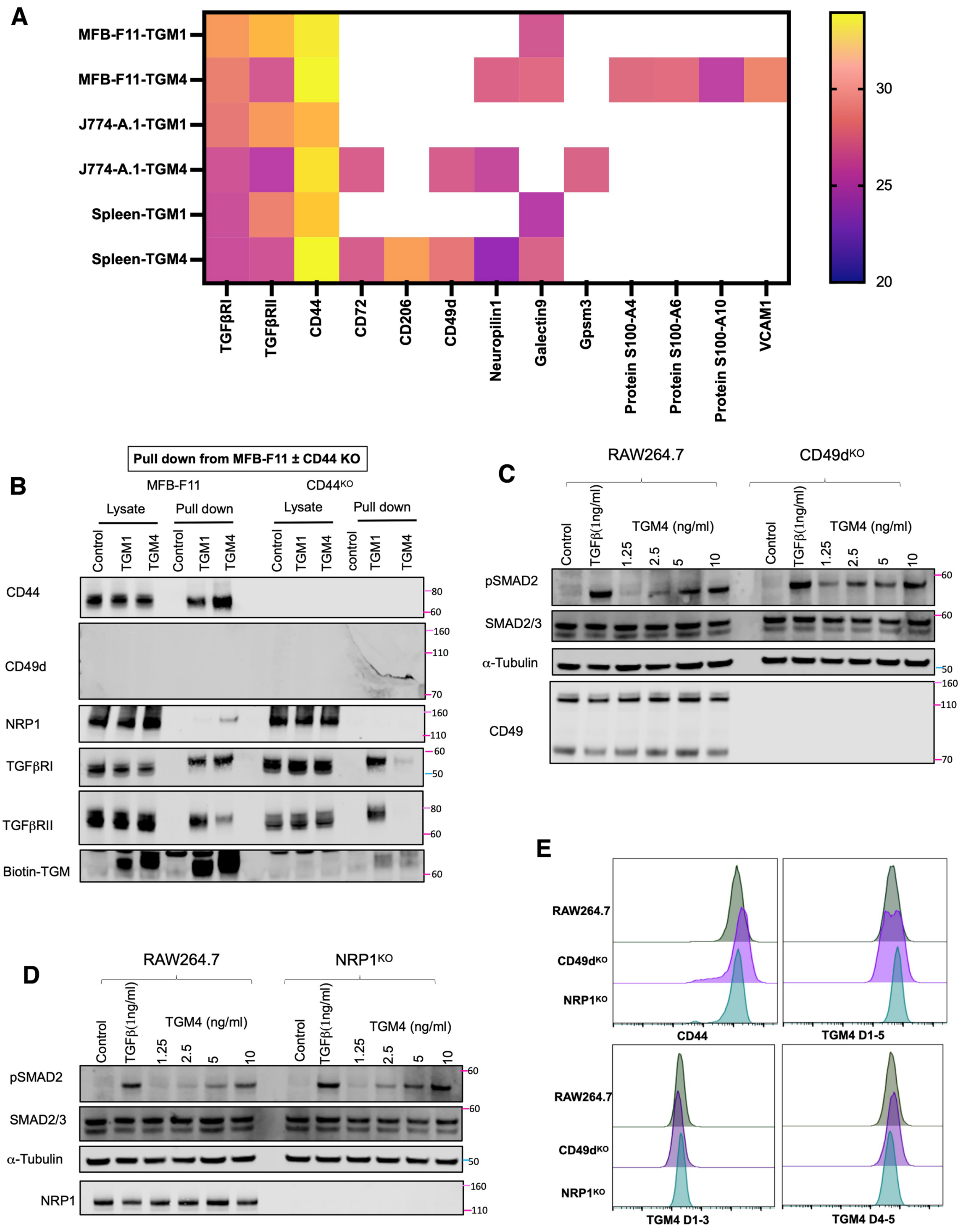
A
MFB-F11-TGM1
MFB-F11-TGM4
J774-A.1-TGM1
J774-A.1-TGM4
Spleen-TGM1
Spleen-TGM4
TGFβRI
TGFβRII
CD44
CD72
CD206
CD49d
Neuropilin1
Galectin9
Gpsm3
Protein S100-A4
Protein S100-A6
Protein S100-A10
VCAM1
30
25
20
B
Pull down from MFB-F11 ± CD44 KO
MFB-F11
CD44KO
Lysate
Pull down
Control
TGM1
TGM4
control
CD44
CD49d
NRP1
TGFβRI
TGFβRII
Biotin-TGM
C
RAW264.7
CD49dKO
TGFβ(1ng/ml)
TGM4 (ng/ml)
1.25
2.5
5
10
pSMAD2
SMAD2/3
α-Tubulin
CD49
D
RAW264.7
NRP1KO
NRP1
E
RAW264.7
CD49dKO
NRP1KO
CD44
TGM4 D1-5
TGM4 D1-3
TGM4 D4-5

Figure EV5. Functional analysis of co-receptors.

(**A**) Heat map of mean mass spectrometry Label-Free Quantitation intensity values for proteins detected in each case, in the samples presented in Fig. 5A–F. (**B**) Pull-down and Western blot analysis in CD44-sufficient and -deficient MFB-F11 cells, probed with antibodies to the indicated proteins. Image is from one of 3 biological replicate experiments. (**C**) SMAD phosphorylation in RAW264.7 cells sufficient or deficient for CD49d, following stimulation with the indicated concentrations of TGM4. Image is from one of 3 biological replicate experiments. (**D**) As (**B**), but with RAW264.7 cells sufficient or deficient for NRP-1. Image is from one of 3 biological replicate experiments. (**E**) Flow cytometric measurement of TGM4 binding to cells lacking CD49d or NRP-1. RAW264.7 control cells, and sublines in which expression of CD49d or NRP-1 was genetically deleted, were probed by flow cytometry for binding to anti-CD44, TGM4 D-15, D1-3 or D4–5 as indicated.

 © The Author(s)
